# Difficult Labors; Aid without the Use of Instruments

**Published:** 1855-04

**Authors:** Reuben Searcy

**Affiliations:** Tuscaloosa, Ala.


### Art. II.—DIFFICULT LABORS; AID WITHOUT THE USE OF INSTRUMENTS.

BY REUBEN SEARCY, M. D., OF TUSCALOOSA, ALA.

No branch of medicine has received more attention than the obstetrical art, which has been carried to such a point of perfection that one cannot hope to add much to its resources. Still it is marked by deficiencies and errors, not only of theory, but of practice, it must be admitted, may yet be detected in it. The experience of every practitioner must have brought under his observation points in which improvement might be made. My own experienee, which has been considerable in this department of the profession, has convinced me that instruments are frequently resorted to in cases of difficult labor where a little manual tact would be found sufficient to accomplish the delivery. I will give the history of a case in which I am convinced such a resort would have been made but for the assistance rendered with the hands.

Mrs. M., aged about 30 years, on the night of October 12th, 1854, was taken in her first labor. On visiting her I found the membranes ruptured, but her pains were slight, returning at long intervals, and felt chiefly in her back. The vertex presented, but the head was not engaged in the upper strait; the os uteri was but little dilated, rigid, and turned very much backward. The bowels being confined I ordered castor oil, which produced the desired effect. The pains continued inefficient, sometimes increasing in force, but again declining, affording the patient an opportunity to sleep in the intervals, and thus the case went on through the 13th and 14th, when the head had descended and was engaged in the superior strait. But in consequence of the retroversion, the parietes of the uterus were receiving the full force of the expulsive efforts. The pains were not severe, but the contractile throes of the uterus were wearing out the strength of the woman and exhausting its own energy. The os was still too rigid, and, for this, by means of my index finger, I applied a small quantity of the extract of belladonna to the part, and on its becoming relaxed drew it forward during the time of a pain. This movement, I found, had the effect of increasing the expulsive force of the womb, and by the morning the head, which had progressed very slowly, was engaged in the lower strait. By applying my fingers on each side of the os coccygis I was now able to reach the head and to aid materially the expulsive efforts of the uterus. The head descending yet lower, the assistance which it was in my power thus to render, was still further increased. Pressing the points of the four fingers of each hand at the extremity of the coccyx, I could exert a pretty decided extractive force, which resulted in the delivery of the child after several pains. The pressure was relaxed on the subsidence of the pain and renewed on each recurrence. The child was healthy and the mother did well,—experiencing no unusual soreness from her labor.

In difficult and protracted labors, when the strength of the woman has begun to fail, and the head of the child has reached the vulva and begins slightly to distend the perinæum, by the application of the fingers as above described, I have found that essential aid may be given to the uterus in its contractile efforts. As the head descends, the fingers may be pressed under the chin of the child, felt through the integuments, and the force necessary to hasten the delivery thus safely and effectually applied. The pressure should be suspended during the intermission of pain, to be renewed on its return. Such pressure is not attended with danger either to mother or child, but on the contrary the life of the latter may be preserved by the prompt delivery, while much suffering is saved to the former. In case of danger to the perinæum, from the pressure of the head of the child, the hand of the accoucheur is in the proper place to afford the necessary support.

*March* 22d, 1855.

